# Plasmid-Driven Resistome Diversity in 9700 *Escherichia coli* Genomes Across Phylogroups and Sequence Types

**DOI:** 10.3390/antibiotics15030287

**Published:** 2026-03-12

**Authors:** Adel Azour, Ghassan M. Matar, Melhem Bilen

**Affiliations:** 1 Department of Experimental Pathology, Immunology, and Microbiology, Faculty of Medicine, American University of Beirut, Beirut 1107 2020, Lebanon; aa630@aub.edu.lb (A.A.); gmatar@aub.edu.lb (G.M.M.); 2 Centre of Infectious Diseases Research, American University of Beirut, Beirut 1107 2020, Lebanon; 3 World Health Organization (WHO) Collaborating Centre for Reference and Research on Bacterial Pathogens, Beirut 1107 2020, Lebanon

**Keywords:** *E. coli*, antimicrobial resistance, plasmidome, resistome, plasmid replicons, multidrug resistance, genomic surveillance

## Abstract

**Background/Objectives**: Plasmids are key vehicles for the dissemination of antimicrobial resistance (AMR), yet their contribution to the global resistome architecture of *Escherichia coli* remains poorly resolved. This study aimed to quantify how plasmid backbones shape the distribution, mobility, and stabilization of resistance genes across diverse phylogenetic backgrounds. **Methods:** We analyze 9700 high-quality genomes spanning major phylogroups and sequence types. Plasmidome reconstruction was integrated with lineage-resolved antimicrobial resistance gene (ARG) mapping to characterize plasmid–ARG associations and evolutionary patterns. **Results:** Although most antimicrobial resistance genes (ARGs) are chromosomal, plasmids disproportionately encode clinically important determinants including *bla*_NDM-5_, *mcr-1.1*, and multiple *bla*_CTX-M_ alleles that show strong, recurrent associations with a restricted set of backbone families, most notably IncX3, IncX4, IncI, and IncF. These conserved plasmid–gene modules recur across phylogenetic backgrounds and continental scales. We identify a marked divergence in evolutionary strategies: generalist phylogroups (A, B1, D) maintain plasmid-rich and highly diverse resistomes, whereas globally dominant Extraintestinal Pathogenic *E. coli* (ExPEC) clones such as ST131 and ST410 exhibit reduced plasmid dependency and frequent chromosomal integration of extended-spectrum β-lactamase (ESBL) genes, particularly bla_CTX-M-15_, consistent with a shift toward vertically stabilized resistomes. By integrating plasmidome reconstruction with lineage-resolved ARG mapping, this study delivers the most extensive plasmid-focused resistome analysis to date, revealing highly modular plasmid–ARG networks structured around a small number of high-risk backbone types. These backbones account for the majority of globally relevant ARGs, including 64.6% of *bla*_NDM-5_ and 76.4% of *mcr-1.1* detections. **Conclusions**: Together, our findings establish plasmid lineages rather than individual genes or clones as central units of AMR dissemination and critical targets for future genomic surveillance and intervention strategies.

## 1. Introduction

Antimicrobial resistance (AMR) has become one of the most pressing global health challenges, driven largely by the rapid emergence and widespread dissemination of resistant bacterial lineages across human, animal, and environmental settings [1]. *Escherichia coli* (*E. coli*) plays a central role in this crisis. As both a ubiquitous commensal and a leading cause of extraintestinal infections, it serves as a key reservoir and vehicle for mobile resistance determinants [2]. Its ability to acquire, maintain, and disseminate antimicrobial resistance genes (ARGs) is tightly linked to its genomic plasticity, particularly the role of plasmids—self-replicating mobile elements that facilitate horizontal gene transfer at scales unmatched by chromosomal evolution [3,4].

Plasmids are well-established vectors for some of the most clinically significant resistance genes, including extended-spectrum β-lactamases (ESBLs), carbapenemases, and colistin resistance genes [5,6]. These include *bla*_CTX-M_ variants, *bla*_NDM_ carbapenemases, and the *mcr* family, whose global dissemination has been strongly linked to specific plasmid families, notably IncF, IncX, IncI, and related types [7,8]. Growing evidence indicates that plasmids and ARGs associations are not random: certain plasmid backbones consistently co-localize with particular resistance genes, forming stable modules that occurs across unrelated lineages and diverse ecological niches [9,10]. These stable associations complicate AMR surveillance, as they facilitate rapid cross-host and cross-clone transmission of high-risk genes.

Despite considerable research on AMR in *E. coli*, most studies have focused on narrow taxonomic or genomic scopes such as individual high-risk clones (e.g., ST131) [11,12], specific resistance genes like *mcr-1* or *bla_CTX-M_* [13,14,15], or geographical confined clinical settings [16]. Recent long-read studies, including the landmark analysis by Arredondo-Alonso et al. [17], reconstructed complete plasmidomes and chromosomes from 2000 extra-intestinal pathogenic *E. coli* (ExPEC) bloodstream isolates collected over two decades. While their work provided key insights into plasmid–host evolutionary dynamics and demonstrated parallel plasmid-mediated and chromosomal strategies for clone success, it focused primarily on clinical clones within a single national cohort. As a result, broader questions about plasmidome diversity and resistome structure across the full phylogenetic and ecological spectrum of *E. coli* remain open.

In contrast, the global architecture of the *E. coli* plasmidome, defined here as the complete repertoire of plasmids circulating within the species, including their diversity, distribution, and associated genetic cargo remains poorly resolved. Plasmids are pivotal to AMR evolution because they mediate horizontal transfer of resistance genes across unrelated lineages, hosts, and environments, allowing *E. coli* to acquire AMR far more rapidly and flexibly than through chromosomal mutation alone. This plasmid-mediated exchange amplifies the complexity of the *E. coli* resistome by linking mobile ARGs, chromosomal integration events, and clonal background into highly dynamic, mosaic genomic architectures. Consequently, key questions remain: how different plasmid families vary in their capacity to mobilize priority ARGs, how plasmid–gene associations differ across phylogroups and sequence types, and how plasmid-driven AMR evolution compares with emerging trends of chromosomal stabilization, such as the increasing integration of ESBL genes within the chromosome [17,18,19,20,21].

Addressing these gaps requires analyses that integrate plasmidome reconstruction, genomic context, and lineage-level population structure at a scale large enough to capture the diversity and evolutionary dynamics of the species. By examining 9700 high-quality *E. coli* genomes, this study provides an extensive plasmid-focused resistome analysis conducted to date. Through this approach, we offer a detailed and global view of how plasmid backbones, chromosomal integration events, and clonal lineages interact to shape AMR evolution in *E. coli*, establishing a foundation for future surveillance strategies centered on plasmid lineages rather than individual resistance genes.

## 2. Results

### 2.1. Plasmid Diversity Across the E. coli Collection

Among the 9700 *E. coli* genomes included in our dataset, 7758 (80%) contained at least one plasmid, representing a total of 24,201 plasmids. Plasmid carriage varied substantially across isolates, ranging from 1 to 13 plasmids per genome. The overall mean was 3.12 plasmids per genome, with a median of 3.0, and most isolates carried between one and five plasmids (Table 1).

When examining plasmid replicon types, we observed that a relatively small set of plasmid families accounted for a large fraction of all plasmids identified. The most abundant replicon was IncFIB(AP001918), with 4311 copies detected, followed by ColRNAI (2387), Col156 (1768), IncFIA (1542), and IncFIC(FII) (1517). Several other families including Col(MG828), IncI1_1_Alpha, IncFII, IncX1, Col440I, IncY, and Col8282 were also well represented (Table 2).

Plasmid burden showed clear differences across phylogroups. Phylogroup C harbored the highest number of plasmids on average (mean = 4.40), followed by G (3.52) and F (3.32). In contrast, groups such as B1, D and E showed lower plasmid loads, while the human-associated groups A and B2 displayed intermediate values (3.13 and 3.26, respectively). These patterns were supported statistically by a Kruskal–Wallis test (H = 363.97, *p* = 6.6 × 10^−73^), and the Dunn post hoc comparisons identified several significant pairwise differences (Table 3).

At the sequence-type (ST) level, plasmid counts were even more variable. A small number of rare STs carried unusually high plasmid loads often eight or nine plasmids per genome despite being represented by single isolates. Examples include ST773, ST7236, ST6775 and ST772 (Table 4).

### 2.2. Phylogroup and ST Variation in Plasmid Load and Composition

**Figure 1:**
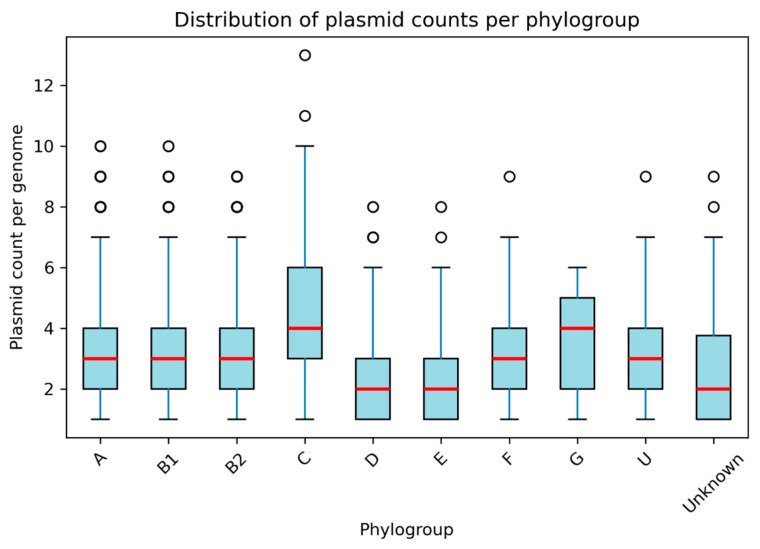
Distribution of plasmid counts per genome across *E. coli* phylogroups. Each boxplot represents the number of plasmids detected per genome within each phylogroup. Red lines indicate median values, boxes show interquartile ranges, and dots represent outliers. Phylogroups C, G and F exhibit the highest plasmid burdens, while D and E show consistently lower values.

Marked variation in plasmid composition was observed across phylogroups (Figure 1).

**Figure 2:**
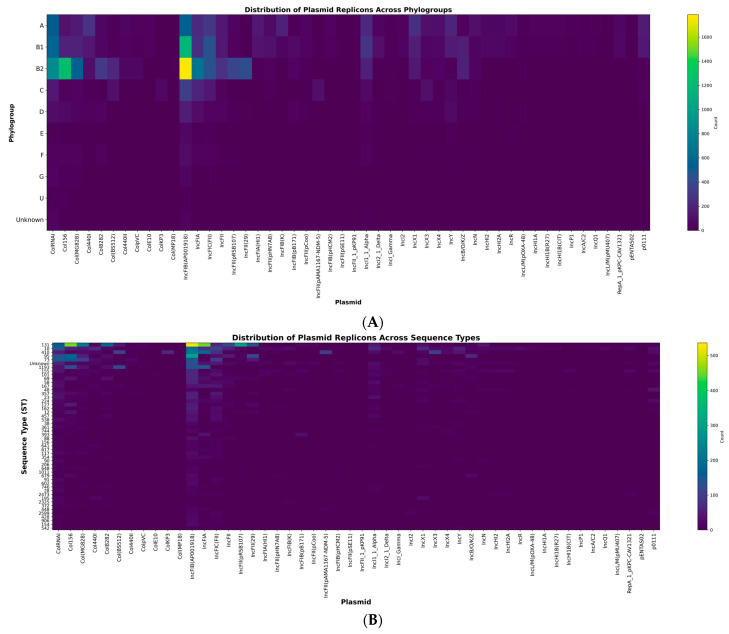
(**A**) Heatmap showing the distribution of plasmid replicon families across the major *E. coli* phylogroups (A, B1, B2, C, D, E, F, G, U and Unknown). Each column represents a distinct plasmid replicon, and each row corresponds to a phylogroup. Color intensity indicates the number of genomes within each phylogroup carrying the corresponding replicon. Phylogroup B2 displays the highest concentration of IncF-type plasmids—including IncFIB(AP001918), IncFIA and IncFIC(FII)—whereas phylogroups A and B1 exhibit higher frequencies of Col-type plasmids such as ColRNAI, Col156 and Col(MG828). Other phylogroups show lower plasmid loads with more diffuse replicon patterns. (**B**) Heatmap illustrating the distribution of plasmid replicon families across common *E. coli* sequence types (STs). Each column represents a plasmid replicon, and each row corresponds to a sequence type. Color intensity reflects the number of isolates within each ST carrying the given replicon. Several dominant STs—including ST131, ST10, ST38 and ST648 harbor a broad set of plasmid replicons, combining IncF, IncI, IncX and Col families, consistent with multi-replicon plasmid carriage. Most other STs show narrower patterns, often dominated by one or a few replicon types.

Phylogroup C carried the highest plasmid burden (mean = 4.40 per genome), followed by G and F, whereas D, E and unassigned isolates exhibited lower plasmid counts. Phylogroup B2 dominated by clinically important lineages such as ST131 displayed a strong enrichment in IncF-type plasmids. In contrast, phylogroups A and B1 showed increased frequencies of Col-type replicons, while phylogroups F and G were characterized by moderate but consistent enrichment in IncF plasmids (Figure 2A).

At the sequence-type level, ST131 showed a distinctive plasmid profile dominated by IncFIB(AP001918), IncFIA and IncFIC(FII). ST10 carried primarily Col-type plasmids, whereas ST38 and ST648 showed profiles enriched in IncX3/IncX4 and mixed IncF/IncI/Col elements, respectively (Figure 2B).

To refine lineage-level differences in plasmid distribution, we quantified the phylogroup and sequence-type representation of the most prevalent plasmid replicons. Col(BS512) was detected in 393 genomes (5.1% of the 7758 plasmid-positive isolates). Its distribution was dominated by phylogroup B2 (213 genomes; 54.2%), followed by A (29 genomes; 7.4%) and B1 (23 genomes; 5.9%). Across these groups, Col(BS512) was subdivided across more than 20 STs, including ST10, ST46, ST167 and ST295 in phylogroups A and B1, and ST12, ST73, ST91, ST131 and ST405 in phylogroup B2; no single ST accounted for more than 6% of all occurrences.

In addition to Col(BS512), several other plasmid families exhibited broad lineage coverage and strong associations with specific sequence types. The IncFIB(AP001918) replicon, detected in 4311 genomes (55.6%), was the most widespread. Its distribution was enriched in phylogroups B2 (34%), B1 (21%), A (18%), F (5%) and C (4%), with the remaining 18% spread across D, E, G and unassigned isolates. At the ST level, this replicon was most common in ST131 (9.4% of all IncFIB-positive genomes), ST10 (7.1%), ST38 (5.5%), ST73 (4.2%), ST648 (3.8%), and ST12 (3.1%), with more than 50 additional STs represented at lower frequency.

The IncI1_1_Alpha plasmid (916 genomes; 11.8%) showed a distinct pattern centered on phylogroups A (27%), B1 (24%) and B2 (18%), followed by F (8%) and C (5%). This plasmid family was distributed across 30 STs, most commonly ST10 (8.5%), ST101 (5.9%), ST155 (4.3%), ST167 (3.7%) and B2-ST131 (3%), with numerous additional STs represented by few isolates.

The IncX1 replicon (668 genomes; 8.6%) was most frequent in phylogroups A (33%), C (20%), and B2 (16%), with additional representation in B1, F and G. Within these groups, IncX1 was primarily associated with ST10, ST744, ST224 and ST101, each contributing 6–11% of all IncX1 occurrences.

Together, these patterns highlight substantial heterogeneity in plasmid lineage coverage.

Some replicons such as IncFIB(AP001918) and Col156 showed broad phylogroup distribution and extensive ST diversification, whereas others such as Col(BS512) and IncI1_1_Alpha exhibited multi-lineage presence but more structured, lineage-dependent profiles.

### 2.3. Distribution of High-Impact ARGs Across Plasmid Replicons

**Figure 3:**
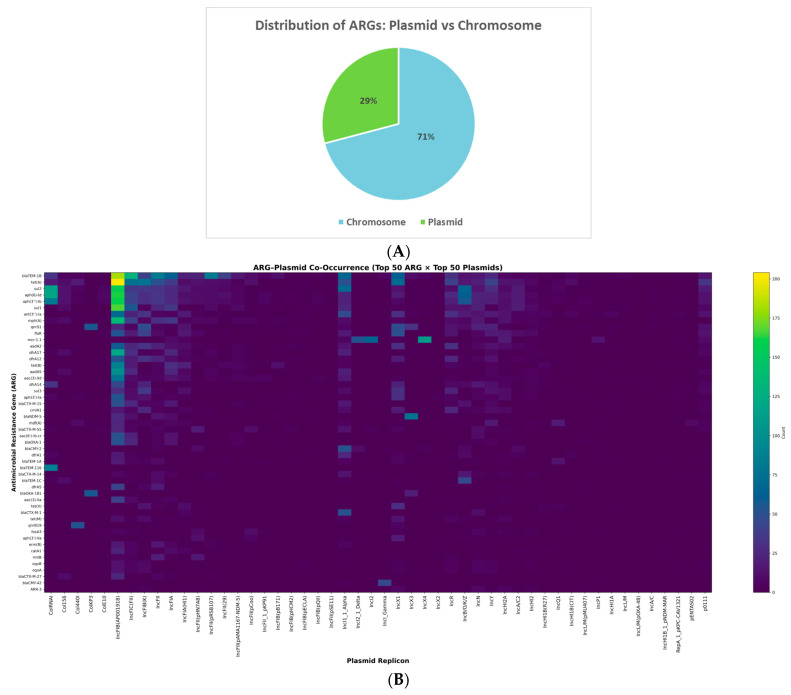
(**A**) Proportion of antimicrobial resistance genes (ARGs) located on plasmids versus chromosomes across the 9700 *E. coli* genomes analyzed. The pie chart shows that 29.1% of all detected ARGs were plasmid-borne, while 70.9% were located on the chromosome. Values represent the percentage of total ARG counts assigned to each genomic compartment. (**B**) Heatmap representing co-occurrence frequencies between the top 50 antimicrobial resistance genes (ARGs) and the top 50 plasmid replicon families. Rows correspond to ARGs and columns to plasmid replicons. Color intensity indicates the number of genomes in which a given ARG–plasmid combination was detected. Distinct vertical concentration patterns are visible for several plasmid families, including IncFIB(AP001918), IncFIA, IncFIC(FII), IncFII and IncI1_1_Alpha, which show the highest ARG co-occurrence levels.

Across the 9700 *E. coli* genomes, a total of 42,826 ARG hits corresponding to 274 distinct genes were identified, including 12,451 (29%) plasmid-borne and 30,375 (71%) chromosomal occurrences (Figure 3A).

At the genome level, 2764 isolates (28.5%) carried at least one plasmid-associated ARG, whereas 6936 genomes (71.5%) encoded their resistome exclusively on the chromosome. Among plasmid-positive isolates, 2637 genomes (27.2%) carried ARGs on both compartments, and 127 genomes (1.3%) harbored all detected ARGs on plasmids.

To characterize the organization of plasmid-associated resistance determinants, we analyzed co-occurrence patterns between the 50 most frequent ARGs and the 50 most abundant plasmid replicons, representing more than 85% of all plasmid-borne ARG occurrences (Figure 3B).

Extended-spectrum β-lactamase (ESBL) genes showed strong and structured associations with IncF-type plasmids. Among plasmid-associated *bla*_CTX-M_ occurrences, IncFIB(AP001918) carried approximately 30%, IncFIA 20%, and IncFIC(FII) 15%, together accounting for 65–70% of all plasmid-borne *bla*_CTX-M_ genes. Within this group, *bla*_CTX-M-15_ represented the dominant allele and was most frequently associated with IncFIB(AP001918) and IncFIC(FII) replicons.

Carbapenemase and colistin resistance genes exhibited even tighter plasmid backbone specialization. Among plasmid-associated *bla*_NDM-5_ occurrences (*n* = 159), 147 (92.6%) were carried by IncX3 plasmids, with the remaining 12 occurrences distributed across a small number of IncF- and IncI-type replicons. Similarly, among plasmid-associated *mcr-1.1* occurrences (*n* = 323), 271 (83.9%) were linked to IncX4, while 48 (14.7%) were carried by IncHI2/IncHI2A plasmids, together accounting for >98% of plasmid-borne *mcr-1.1*.

Other frequently mobilized resistance determinants formed broader MDR modules. Among plasmid-associated *sul2* occurrences (*n* = 1259), Col156, IncFIA, IncFIB(AP001918) and IncI1_1_Alpha collectively carried 780 occurrences (62%). For *tet*(*A*), IncI1_1_Alpha alone accounted for 27% of plasmid-borne copies (200 of 728), followed by IncFII and several Col-type replicons. *aadA*-family genes showed a similar pattern, with IncFIB(AP001918), IncFIC(FII), IncI1_1_Alpha and IncI2 jointly carrying 65% of the 859 plasmid-associated *aadA* detections.

Plasmid-mediated quinolone resistance genes were less abundant but highly mobile. Among plasmid-associated *qnrS*-family genes (*n* = 663), IncFIA, IncFIB(AP001918) and IncI1-type plasmids together accounted for 70% of occurrences, indicating recurrent recruitment of *qnrS* into IncF- and IncI-associated MDR modules.

These data demonstrate that plasmid-borne ARGs are concentrated within a limited number of replicon families, which collectively account for most high-impact resistance genes.

### 2.4. MDR-Associated Plasmid Profiles in Major Sequence Types

**Figure 4:**
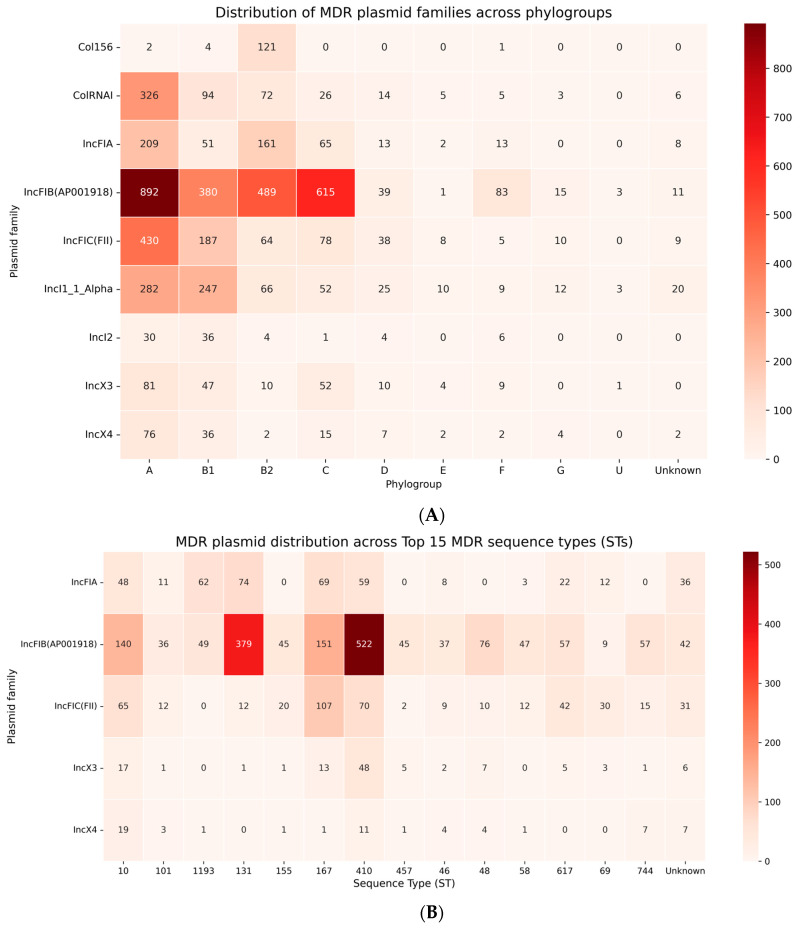
(**A**) Distribution of MDR-associated plasmid families across phylogroups. Heatmap showing the number of plasmid occurrences for the major MDR–associated replicon families (IncFIA, IncFIB(AP001918), IncFIC(FII), IncI1_1_Alpha, IncX3, IncX4) across *E. coli* phylogroups. Counts represent the total number of plasmid–genome observations per phylogroup. IncF-type plasmids dominate phylogroups B2 and C, whereas Col- and IncI-type plasmids are more frequent in phylogroups A and B1. Color intensity reflects plasmid frequency, with darker shades indicating higher abundance. (**B**) Distribution of MDR-associated plasmid families across the Top 15 MDR sequence types (STs). Heatmap showing plasmid family occurrences across the sequence types most enriched in MDR-associated plasmids. IncFIB(AP001918) and IncFIC(FII) are strongly concentrated in ST131, ST410, ST167 and ST48, whereas IncX3 and IncX4 are predominantly associated with carbapenemase- and colistin-linked lineages such as ST48, ST744, ST410 and ST46. Each cell indicates the number of isolates carrying the corresponding plasmid family within each ST. Color intensity reflects plasmid frequency.

Marked heterogeneity in MDR-associated plasmid carriage was observed across phylogroups and sequence types (Figure 4A).

Among the six major MDR plasmid families, IncFIB(AP001918) was by far the most abundant, with 2460 occurrences, followed by IncFIC(FII) (758), IncI1_1_Alpha (728), IncFIA (512), IncX3 (212), and IncX4 (146). At the phylogroup level, B2 carried the largest MDR plasmid burden, including 892 IncFIB(AP001918) replicons (36.3% of all B2 plasmid observations), 430 IncFIC(FII) replicons (17.5%), and 282 IncI1_1_Alpha plasmids (11.5%). Phylogroup C also exhibited high MDR plasmid density, dominated by 615 IncFIB(AP001918) occurrences, together with 78 IncFIC(FII) and 52 IncI1_1_Alpha replicons. In contrast, phylogroups A and B1 carried fewer MDR plasmids but showed broad diversity, with A harboring 326 ColRNAI, 209 IncFIA, and 430 IncFIC(FII) plasmids, while B1 contained 380 IncFIB(AP001918), 247 IncI1_1_Alpha, and 187 IncFIC(FII) plasmids. These data illustrate that MDR plasmids are not restricted to a single phylogroup: although B2 remains the principal hotspot, substantial MDR plasmid reservoirs also exist within A, B1, and C.

At the sequence-type level, MDR plasmid families displayed strikingly unequal distributions (Figure 4B).

ST410 (phylogroup C) represented the most significant MDR hub, carrying 522 IncFIB(AP001918) plasmids (21.2% of all IncFIB detections worldwide), together with 70 IncFIC(FII) plasmids (9.2% of total IncFIC detections) and 59 IncFIA replicons (11.5%), confirming its central role in ESBL-associated plasmid dissemination. ST131 (phylogroup B2) constituted the second major MDR lineage, with 379 IncFIB(AP001918) replicons (15.4% of the global total), 12 IncFIC(FII) plasmids (1.6%), and 74 IncFIA replicons (14.5%), consistent with its well-established association with IncF-mediated resistance.

ST167 (phylogroup A) carried a substantial MDR plasmid load, including 151 IncFIB(AP001918) plasmids (6.1%), 107 IncFIC(FII) plasmids (14.1%), and 69 IncFIA replicons (13.5%), reflecting its importance as a broad-spectrum MDR lineage. ST410 further accumulated carbapenemase- and colistin-associated plasmids, including 48 IncX3 replicons (22.6% of all IncX3 detections) and 11 IncX4 plasmids (7.5%), highlighting its additional role in last-resort resistance dissemination.

ST10 (phylogroup A), although highly prevalent, displayed a more commensal-like profile with moderate MDR involvement (140 IncFIB(AP001918), 65 IncFIC(FII), and 48 IncFIA plasmids), while ST744 showed moderate IncFIB(AP001918) (57) and IncX4 (7) representation. Altogether, these quantitative patterns demonstrate that MDR plasmid dissemination is dominated by a limited number of plasmid–ST partnerships, with ST410 and ST131 representing the principal IncFIB-associated hubs and IncX3/IncX4 plasmids being largely concentrated in ST410 and selected additional lineages. This structured, non-random distribution underscores the presence of high-impact sequence types that act as central conduits for plasmid-mediated AMR dissemination across the *E. coli* population.

### 2.5. Co-Occurrence Patterns Between Plasmids and Resistance Genes

**Figure 5:**
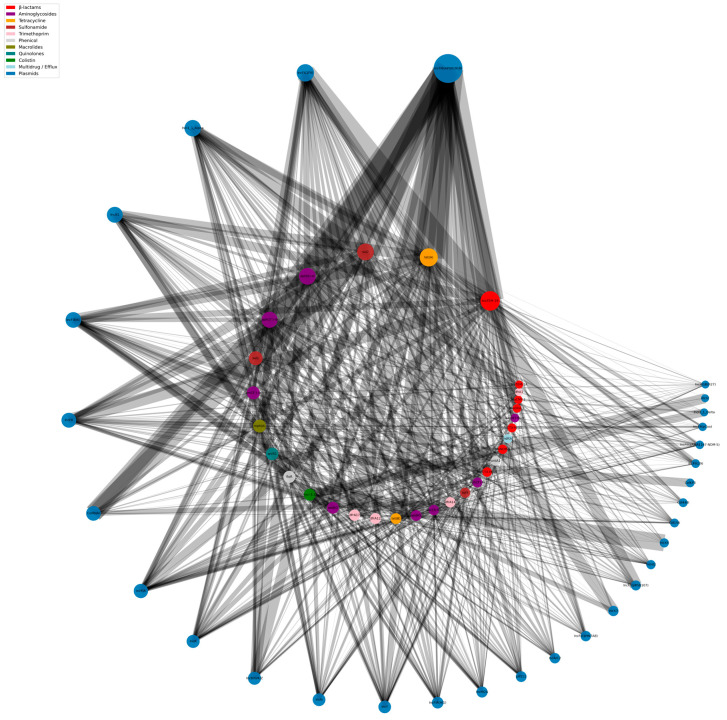
Network representation of plasmid–ARG co-occurrence patterns across the *E. coli* dataset. Each node represents either a plasmid replicon (blue nodes) or an antimicrobial resistance gene category (colored nodes according to antibiotic class). Edges indicate co-occurrence within the same genome, with edge density proportional to the number of genomes sharing the association. Highly connected plasmid nodes, including IncF and IncI families, cluster near the center of the network, while less frequent plasmid families appear at the periphery.

ARG–plasmid co-occurrence analyses confirmed that a small number of plasmid families act as major hubs of the plasmid-borne resistome (Figure 5).

IncFIB(AP001918) was the largest ARG carrier, with 2378 ARG occurrences mapped to this replicon (19.1% of all plasmid-borne ARG hits). The most frequent co-occurring genes included *sul2* (448 occurrences; 18.8% of the IncFIB-associated resistome), *tet*(*A*) and its variants (358; 15.1%), *aadA*-type aminoglycoside-modifying enzymes (279; 11.7%), *bla*_TEM-1B_ (210; 8.8%) and *bla*_CTX-M-15_ (146; 6.1%). This dense cluster of sulfonamide, tetracycline, aminoglycoside and ESBL determinants around IncFIB(AP001918) forms a core MDR module.

IncFIA and IncFIC(FII) plasmids displayed similar, though smaller, MDR modules. IncFIA carried 874 ARG occurrences, enriched in *sul2* (173; 19.8%), *tet*(*A*) variants (121; 13.8%), *aadA* genes (98; 11.2%), *bla*_TEM-1B_ (74; 8.5%) and *bla*_CTX-M-15_ (58; 6.6%). IncFIC(FII) harbored 477 ARG occurrences, again dominated by *sul2* (92; 19.3%), *tet*(*A*) (63; 13.2%), *aadA* (54; 11.3%) and *bla*_TEM-1B_ (39; 8.2%). Together, these three IncF families formed a large, interconnected module comprising ESBLs, aminoglycoside-modifying enzymes, sulfonamide and tetracycline resistance genes.

IncI1_1_Alpha formed a second major MDR module. This replicon carried 799 ARG occurrences, frequently including *bla*_CTX-M-15_ (118; 14.8%) and *bla*_TEM-1B_ (97; 12.1%), together with *sul2* (119; 14.9%), *aadA* variants (89; 11.1%) and *tet*(*A*) (68; 8.5%). This pattern supports a role for IncI1 in mobilizing ESBLs alongside aminoglycoside and tetracycline resistance.

By contrast, IncX-type plasmids were more specialized. IncX3 carried 158 ARG occurrences, dominated by *bla*_NDM-5_ (116; 73.4%), with additional co-carriage of other β-lactamase, aminoglycoside and sulfonamide genes at much lower frequencies. IncX4 (256 ARG occurrences) was strongly associated with *mcr-1.1* (205; 80.1%), with remaining ARGs predominantly involving *tet* and *sul* genes. These two plasmid families formed a distinct module linking carbapenemase (IncX3–*bla*_NDM-5_) and colistin (IncX4–*mcr-1.1*) determinants to a narrower accessory resistance background.

Col-type plasmids contributed smaller, more peripheral clusters. Col156, ColRNAI and Col(MG828) were most frequently associated with *tet*(*A*), *sul2* and *aadA* variants, but only rarely with ESBLs or carbapenemases. Overall, the network structure highlighted a few highly connected MDR hubs (IncF- and IncI-type plasmids) and more specialized vehicles for last-resort resistance genes (IncX3 and IncX4).

### 2.6. Comparative Contribution of Plasmids and Chromosomes to the Resistome

Plasmid and chromosomal compartments contributed unevenly to the carriage of individual resistance genes. While most ARGs remained primarily chromosomal, several clinically important determinants showed strong plasmid enrichment. For example, *bla*_NDM-5_ was detected 246 times, of which 159 (64.6%) were located on plasmids mostly IncX3 while the remaining 87 (35.4%) were chromosomal. *mcr-1* variants (mostly *mcr-1.1*) showed an even stronger plasmid bias, with 323 of 423 detections (76.4%) were located on plasmids, predominantly IncX4 replicons.

In contrast, *bla*_CTX-M-15_ displayed a more mixed distribution, with 757 total occurrences split between plasmids (169; 22.3%) and chromosomes (588; 77.7%). Plasmid-mediated quinolone resistance genes of the *qnrS* family were predominantly plasmid-borne (663/809 detections; 82.0%), while *sul2* (1259/2474; 50.9%) and *aadA* variants (859/1377; 62.4%) showed intermediate plasmid contributions. The efflux pump *mdf*(*A*) was largely chromosomal, with only 140 of 9675 detections (1.4%) found on plasmids.

These data highlight that, although the majority of ARGs are chromosomally encoded, plasmids disproportionately contribute to the dissemination of key acquired determinants particularly *bla*_NDM-5_, *mcr-1*, *qnrS* and many aminoglycoside- and sulfonamide-resistance genes.

### 2.7. ARG Burden and Diversity Across Plasmid Families

**Figure 6:**
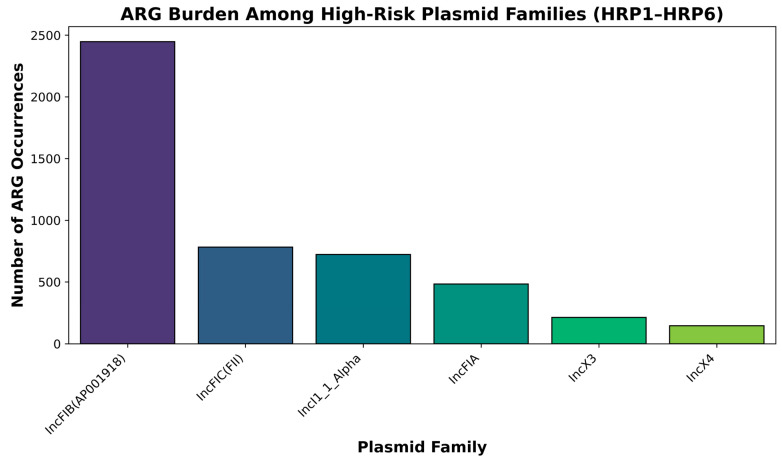
ARG burden among High-Risk Plasmid (HRP) families. Barplot showing the number of antimicrobial resistance gene (ARG) occurrences associated with the six High-Risk Plasmid families (HRP1–HRP6). IncFIB(AP001918) (HRP1) exhibited the highest ARG burden, carrying approximately 2400 resistance determinants across the dataset. IncFIC(FII) and IncI1_1_Alpha (HRP2–HRP3) showed intermediate levels of ARG carriage, predominantly including ESBLs, tetracycline resistance genes, and aminoglycoside-modifying enzymes. IncFIA (HRP4) carried a moderate but diverse ARG set, while IncX3 (HRP5) and IncX4 (HRP6) were enriched for carbapenemase (*bla*_NDM_) and colistin resistance (*mcr-1*) genes, respectively. Together, these plasmid groups represent the core backbones disproportionately contributing to MDR dissemination across the collection.

Plasmid families differed not only in abundance but also in the size and diversity of their ARG cargo. IncFIB(AP001918) carried the largest ARG burden, with 2378 plasmid-borne ARG occurrences spanning 148 distinct genes, representing 19.1% of all plasmid-associated ARG hits in the dataset (Figure 6).

IncFIA and IncFIC(FII) collectively contributed an additional 1351 occurrences across 113 and 90 distinct genes, respectively, reinforcing the central role of IncF-type plasmids as broad-spectrum MDR backbones.

IncI1_1_Alpha harbored 799 ARG occurrences representing 104 distinct genes, many of which were ESBLs (notably *bla*_CTX-M-15_ and *bla*_TEM-1B_), together with sulfonamide, tetracycline and aminoglycoside resistance determinants. IncX3 and IncX4 carried smaller but highly focused ARG repertoires: 158 occurrences across 30 distinct genes for IncX3 (dominated by *bla*_NDM-5_), and 256 occurrences across 33 genes for IncX4 (dominated by *mcr-1.1*).

Col-type plasmids, although highly prevalent, carried more modest and specialized ARG payloads. Col156, ColRNAI and Col(MG828) each contributed several hundred ARG occurrences, largely restricted to *tet*(*A*), *sul1*/*sul2* and streptomycin-resistance genes (e.g., *aph*(*3*″)*-Ib*, *aph*(*6*)*-Id*), and only rarely encoded β-lactamases or carbapenemases. Overall, this pattern indicates that IncF-, IncI- and IncX-type plasmids carry the bulk of clinically important MDR determinants, whereas Col-type plasmids primarily act as vehicles for older, non–β-lactam resistance genes.

### 2.8. Identification and Quantitative Characterization of High-Risk Plasmid (HRP) Groups

**Figure 7:**
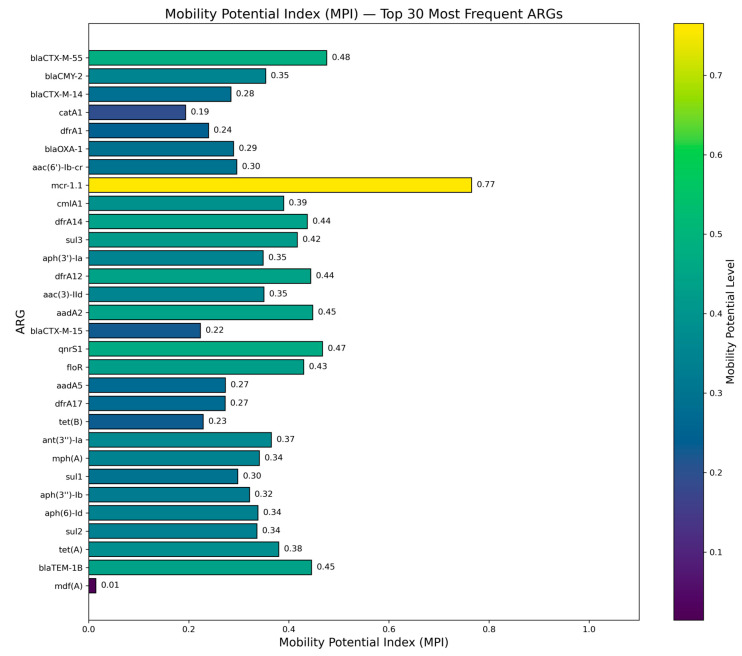
Mobility Potential Index (MPI) among the 30 most frequent antimicrobial resistance genes (ARGs). Horizontal barplot showing the MPI score, defined as the proportion of occurrences of each ARG that were plasmid-borne. A subset of determinants exhibited high mobility potential, including *mcr-1.1* (MPI = 0.77), *aadA2* (0.45), *bla*_TEM-1B_ (0.45), *tet*(*A*) (0.38), *qnrS1* (0.47), *sul3* (0.42), *catA1* (0.19), and multiple *dfrA* variants (MPI = 0.24–0.44). Conversely, chromosomally encoded or low-mobility genes such as *mdf*(*A*) (MPI = 0.01) showed almost no plasmid association. The gradient color scale reflects increasing mobility potential from low (chromosomal) to high (plasmid-enriched). These data highlight strong heterogeneity in gene mobility, underscoring the central role of plasmids in the dissemination of key ARG classes.

Based on combined criteria of abundance, ARG burden and lineage breadth, six plasmid families were classified as High-Risk Plasmid (HRP) groups: IncFIB(AP001918), IncFIA, IncFIC(FII), IncI1_1_Alpha, IncX3 and IncX4 (Figure 7).

Together, these families were detected in 5027 of the 7758 plasmid-positive genomes (64.8%) and carried 4965 plasmid-borne ARG occurrences (39.9% of all plasmid-associated ARG hits).

IncFIB(AP001918) was the most widespread HRP, present in 4311 genomes (55.6% of plasmid-positive isolates), spanning eight phylogroups and 229 distinct STs. IncFIA and IncFIC(FII) were detected in 1542 (19.9%) and 1517 (19.6%) genomes, respectively, each spanning all major phylogroups and >150 STs. IncI1_1_Alpha occurred in 916 genomes (11.8%), across seven phylogroups and 146 STs, while IncX3 and IncX4 were detected in 222 (2.9%) and 301 (3.9%) genomes, respectively, yet remained widely distributed across phylogroups and sequence types.

In terms of ARG content, IncFIB(AP001918) alone carried 2378 ARG occurrences, followed by IncFIA (874), IncI1_1_Alpha (799), IncFIC(FII) (477), IncX4 (256) and IncX3 (158). Each HRP family thus combined (i) high prevalence, (ii) broad phylogroup and ST coverage and (iii) substantial and often clinically critical ARG cargo, justifying their designation as high-risk vehicles for MDR dissemination.

### 2.9. ARG Mobility Potential Index (MPI)

To quantify the propensity of individual ARGs to be carried on plasmids, we calculated a Mobility Potential Index (MPI) for each gene, defined as the proportion of its occurrences found on plasmids. MPI values ranged from near-zero (predominantly chromosomal) to >0.8 (strong plasmid bias).

Among high-impact determinants, *bla*_NDM-5_ displayed an MPI of 0.65 (159/246 occurrences on plasmids), reflecting frequent plasmid association but also a substantial chromosomal component. *mcr-1* variants (mostly *mcr-1.1*) showed a higher MPI of 0.76 (323/423 plasmid-borne), while *qnrS*-family genes were strongly plasmid-associated with an MPI of 0.82 (663/809 occurrences on plasmids). In contrast, *bla*_CTX-M-15_ exhibited a more balanced distribution with an MPI of 0.22 (169/757 plasmid-borne), indicating that in this dataset a majority of *bla*_CTX-M-15_ copies are chromosomally encoded.

Intermediate MPI values were observed for widely distributed accessory genes such as *sul2* (0.51; 1259/2474 plasmid-borne), *aadA* variants (0.62; 859/1377) and *tet*(*A*)-like genes (0.38; 909/2393). The efflux pump *mdf*(*A*), by contrast, had a very low MPI of 0.015, with only 140 of 9675 detections located on plasmids, consistent with its role as a core chromosomal determinant.

Taken together, these MPI profiles indicate that a subset of ARGs particularly *qnrS*, *mcr-1* and *bla*_NDM-5_ are strongly enriched on plasmids and therefore have high potential for horizontal dissemination, whereas others such as *mdf*(*A*) and several native β-lactamase variants remain largely chromosomal and are less mobilization-prone.

## 3. Discussion

This study provides the first plasmidome-resolved analysis of AMR architecture across *E. coli* at a truly global scale, integrating 9700 high-quality genomes spanning all major phylogroups and dominant MDR sequence types. By unifying plasmid backbone diversity, ARG localization, lineage structure, and mobility potential, our work demonstrates that plasmid lineages, not bacterial clones, or individual resistance genes constitute the primary organizational units shaping AMR dissemination in *E. coli*. The global resistome is not diffuse or stochastic; rather, it is structured around a restricted set of hyper-successful plasmid families that repeatedly assemble with the same high-risk resistance determinants. These findings reveal a modular, evolutionarily conserved plasmid–gene architecture that redefines how AMR emerges, spreads, and stabilizes across ecological and geographical boundaries [22,23].

The recurrent associations observed between IncX3–*bla*_NDM-5_, IncX4–*mcr-1.1*, and multiple IncF backbones with *bla*_CTX-M_ alleles provide strong evidence for co-adapted plasmid–gene compatibility modules. These modules persist across unrelated phylogroups and hundreds of sequence types, suggesting that plasmids impose structural constraints that shape the evolutionary landscape of AMR. Such constraints likely arise from backbone-specific replication systems, addiction modules, conjugation machinery, and compensatory evolution that collectively optimize the stability and transmissibility of particular gene combinations [24,25,26,27,28]. This restricted evolutionary “design space” helps explain why AMR dissemination converges on a small number of plasmid backbones despite the enormous genomic diversity of *E. coli*. Our findings extend and globalize observations from long-read clinical studies, showing that these modules are not confined to specific outbreaks or national cohorts; they represent globally persistent evolutionary units that underpin AMR flow across the One-Health continuum [17].

The marked contrast between plasmid-rich resistomes in generalist phylogroups (A, B1, D) and chromosomal stabilization of ESBL determinants in pandemic clones like ST131 and ST410 highlights distinct evolutionary strategies for long-term success. Generalist lineages appear to rely on highly mobile plasmidome architectures that facilitate horizontal exchange and ecological versatility, whereas globally dominant ExPEC clones increasingly stabilize key ESBL genes within the chromosome to reduce plasmid fitness costs and ensure vertical persistence. This duality parallels prior evolutionary models and population structure observations and is consistent with reports of extensive chromosomal fixation of *bla*_CTX-M-15_ in ST131 and ST410 [18,21,29,30,31].

The discovery of stable plasmid–gene modules has direct translational potential. Because these modules recur predictably across lineages and continents, they represent structurally coherent targets for plasmid-focused AMR mitigation strategies. CRISPR-based antimicrobials, for example, could be engineered to selectively disrupt high-risk plasmid families such as IncX3, IncX4, and IncFIB(AP001918), thereby eliminating *bla*_NDM-5_, *mcr-1.1*, or *bla*_CTX-M_ reservoirs at their backbone source rather than targeting individual alleles. The consistency of these plasmid–gene architectures suggests that plasmid-targeted interventions may bypass the formidable genetic diversity of *E. coli*, focusing instead on the limited set of vehicles that sustain global AMR transmission [6].

The structured nature of the plasmidome revealed here provides a foundation for developing plasmid-centric AMR surveillance systems. Instead of tracking hundreds of resistance genes across thousands of clones, genomic surveillance programs can monitor a small set of high-risk plasmid backbones that account for the majority of clinically important ARGs. Plasmidome signatures such as the presence of IncX3, IncX4, or IncFIB(AP001918) could serve as early warning indicators of emerging threats, enabling predictive modeling of AMR dissemination across human, animal, and environmental reservoirs. This plasmid-centered perspective aligns with global sewage surveys [32] and environmental resistome studies [33,34], which similarly highlight recurrent plasmid-driven AMR modules as ecological sentinels.

Collectively, our results advance a new conceptual framework for AMR evolution in *E. coli*: plasmid backbones, not bacterial genomes, constitute the fundamental units driving the global dissemination of resistance. The resistome emerges through modular, lineage-agnostic plasmid–gene combinations that recur across phylogroups, enabling rapid cross-host and cross-ecosystem transmission. This represents a shift from traditional clone-centric models toward a plasmidome-centric understanding of AMR biology. By mapping this architecture at scale, we provide the first global reference framework capable of guiding surveillance, prediction, and intervention strategies targeting plasmid-mediated resistance.

Despite reliance on replicon-based plasmid inference which, may overlook cryptic or highly divergent plasmids the remarkably consistent plasmid–gene associations observed across this extensive dataset underscore the robustness of our conclusions. As long-read sequencing, metagenomics, and functional plasmid biology continue to expand, the plasmidome map generated here will serve as a foundational resource for decoding mobile genetic element evolution, validating plasmid–gene compatibility mechanisms, and designing plasmid-targeted AMR mitigation tools [32,35].

## 4. Materials and Methods

### 4.1. Genome Collection and Quality Filtering

A total of 12,053 *E. coli* genomes were retrieved from the BV-BRC (Bacterial and Viral Bioinformatics Resource Center, Birmingham, AL, USA) database (https://www.bv-brc.org/, accessed on 5 August 2025). The dataset included both complete genomes and high-quality WGS assemblies, as classified in the BV-BRC database. Species identity and taxonomic consistency were verified using FastANI v1.33 (Jain et al., Berkeley, CA, USA) [36], and only genomes showing ≥95% average nucleotide identity to the *E. coli* type strain were retained. After applying additional quality filters based on genome size (4.5–5.5 Mb), GC content (50–51.5%), contig number (≤100), and removal of duplicate entries, 9700 high-quality assemblies were kept for downstream analyses.

The complete list of accession numbers for all 9700 genomes included in this study is provided in Supplementary Table S1 to ensure full transparency and reproducibility.

### 4.2. Genomic Analyses

Plasmid replicons were identified using PlasmidFinder v2.1 (Center for Genomic Epidemiology, Kongens Lyngby, Denmark) (≥95% identity, ≥80% coverage) [37], and ARGs were detected with ResFinder v4.7.2 (Center for Genomic Epidemiology, Kongens Lyngby, Denmark) (≥90% identity, ≥80% coverage) [38]. To prevent inflation of plasmid counts due to assembly or annotation artefacts, replicon hits repeated on the same contig were collapsed, and when multiple replicons overlapped, the one with the higher percentage identity was retained, following the approach used by Acman et al. [39]. The genomic location of each ARG was assigned by intersecting plasmid replicon coordinates with gene annotations. ARGs located on contigs carrying at least one plasmid replicon hit were classified as plasmid-associated; all other contigs were classified as chromosomal. Because plasmid assignment was performed at the replicon and contig level rather than through complete plasmid reconstruction, our approach focuses on backbone detection and ARG co-localization, reducing sensitivity to plasmid fragmentation in draft assemblies.

Phylogroups were determined with EzClermont v0.4.6 (Clermont et al., Paris, France) [40] and sequence types (STs) were assigned using the Achtman MLST scheme [41]. All plasmid-positive genomes, representing 7758 isolates and 24,201 plasmid replicons, were merged with phylogroup, ST and ARG metadata to construct plasmid–gene association matrices.

Multidrug-resistant (MDR) isolates were defined as genomes harboring resistance determinants associated with at least three distinct antimicrobial classes, in accordance with established international definitions [42].

### 4.3. Statistical Analyses

Plasmid load, replicon diversity, and distribution across phylogenetic backgrounds were quantified using Python v3.12.3. Co-occurrence analyses between plasmids and ARGs, as well as class-level enrichment patterns, were derived from frequency tables. The Mobility Potential Index (MPI) of each ARG was calculated as the proportion of plasmid-borne occurrences relative to total occurrences. High-Risk Plasmid (HRP) groups were defined using combined criteria of ARG diversity, presence across multiple phylogroups and STs, and high connectivity in ARG–plasmid bipartite networks reconstructed with NetworkX (NetworkX Developers, Fort Lauderdale, FL, USA). Statistical differences in plasmid burden among phylogroups were assessed using Kruskal–Wallis tests followed by Dunn’s post hoc comparisons with Bonferroni correction (SciPy (SciPy Community, Minneapolis, MN, USA), statsmodels (Statsmodels Developers, USA)). All visualizations, including heatmaps, plasmid–ARG networks, class enrichment barplots, and MPI distributions, were generated in Python (Python Software Foundation, Wilmington, DE, USA).

## 5. Conclusions

Together, our analyses demonstrate that AMR dissemination in *E. coli* is strongly structured around a restricted set of plasmid backbones rather than driven by random gene acquisition. While most ARG occurrences were chromosomal, plasmids disproportionately carried clinically important acquired determinants and formed recurrent backbone–gene compatibility modules, including IncX3–*bla*_NDM-5_, IncX4–*mcr-1.1*, and broad MDR clusters dominated by IncF- and IncI-type plasmids. Six High-Risk Plasmid (HRP) groups emerged as central hubs of plasmid-borne AMR, providing a practical framework for plasmid-focused genomic surveillance. Overall, these findings support plasmidome-centric monitoring as a scalable and predictive strategy to anticipate and track AMR spread across lineages and ecological settings.

## Figures and Tables

**Table 1:** Distribution of plasmid counts among 7758 plasmid-bearing genomes.

Plasmid Count	Number of Genomes
1	1626
2	1636
3	1702
4	1141
5	825
6	482
7	207
8	96
9	35
10	6
11	1
13	1

**Table 2:** Most abundant plasmid replicon types detected in the 7758 plasmid-positive genomes.

Rank	Plasmid Type	Count	% of Total (24,201)
1	IncFIB (AP001918)	4311	17.8%
2	ColRNAI	2387	9.9%
3	Col156	1768	7.3%
4	IncFIA	1542	6.4%
5	IncFIC(FII)	1517	6.3%
6	Col(MG828)	1114	4.6%
7	IncI1_1_Alpha	916	3.8%
8	IncFII	688	2.8%
9	IncX1	668	2.8%
10	Col440I	617	2.5%
11	IncY	600	2.5%
12	Col8282	575	2.4%
13	IncFII(29)	508	2.1%
14	IncB/O/K/Z	463	1.9%
15	p0111	423	1.7%
16	IncX3	363	1.5%
17	IncFIA(HI1)	314	1.3%
18	IncHI2	175	0.7%
19	IncHI2A	171	0.7%
20	IncR	168	0.7%

**Table 3:** Mean plasmid counts across phylogroups.

Phylogroup	Mean	Median	Min	Max	*n* Genomes
C	4.40	4	1	13	419
G	3.52	4	1	6	86
F	3.32	3	1	9	171
B2	3.26	3	1	9	2611
A	3.13	3	1	10	1649
U	2.98	3	1	9	40
B1	2.87	3	1	10	2016
D	2.52	2	1	8	495
Unknown	2.48	2	1	9	122
E	2.40	2	1	8	149

**Table 4:** Top STs with the highest plasmid burden.

ST	Mean Plasmids	Median	Min	Max	*n* Genomes
773	9.0	9	9	9	1
7236	9.0	9	9	9	1
6775	9.0	9	9	9	1
772	8.5	8.5	8	9	2
6833	8.0	8	8	8	1
12,455	8.0	8	8	8	1
4656	8.0	8	8	8	1
5387	7.0	7	7	7	1
8565	7.0	7	7	7	1
7940	7.0	7	7	7	1

## Data Availability

The *E. coli* genomes analyzed in this study are publicly available from the BV-BRC database (https://www.bv-brc.org/) accessed on 5 August 2025.

## Supplementary Materials

The following supporting information can be downloaded at: https://www.mdpi.com/article/10.3390/antibiotics15030287/s1, Table S1: Genome ID.
